# Neuroprotective Effect of *ent*-Kaur-15-en-17-al-18-oic Acid on Amyloid Beta Peptide-Induced Oxidative Apoptosis in Alzheimer’s Disease

**DOI:** 10.3390/molecules25010142

**Published:** 2019-12-29

**Authors:** Caiyun Zhang, Xingming Zhao, Shiqi Lin, Fangyuan Liu, Jiahui Ma, Zhuo Han, Fujuan Jia, Weidong Xie, Qian Zhang, Xia Li

**Affiliations:** 1 Marine College, Shandong University, Weihai 264209, Shandong, China; caiyun617@outlook.com (C.Z.); Xingming1996@outlook.com (X.Z.); lsqsd@outlook.com (S.L.); sdumjh@hotmail.com (F.L.); sdumjh@hotmail.com (J.M.); hanzhuo1013@gmail.com (Z.H.); jfj1996@outlook.com (F.J.); wdxie@sdu.edu.cn (W.X.); zhangqianzq@sdu.edu.cn (Q.Z.); 2 School of Pharmaceutical Sciences, Shandong University, Jinan 250012, China

**Keywords:** Alzheimer’s disease, oxidative stress, apoptosis, Aβ_25-35_, SH-SY5Y, p53, NF-κB

## Abstract

*ent*-Kaur-15-en-17-al-18-oic acid, extracted from the Chinese well known folk herb *Leontopodium longifolium*, performed a significantly neuroprotective effect on amyloid beta peptide 25-35 (Aβ_25-35_)-induced SH-SY5Y cells neurotoxicity in Alzheimer’s disease. The results demonstrated that this compound maintained oxidative stress balance, reduced levels of reactive oxygen species (ROS), malondialdehyde (MDA), and improved contents of glutathione (GSH) and superoxide dismutase (SOD) without obvious cytotoxicity. This compound also obviously relieved oxidative stress-induced apoptosis associated with p53 and nuclear factor κB (NF-κB) pathways accompanied by upregulating B-cell lymphoma-2 (bcl-2) and downregulating p53, nuclear factor κB (NF-κB), Bax, Cleaved-caspase 3, and Cytochrome C protein expressions further. Briefly, *ent*-kaur-15-en-17-al-18-oic acid protected cells from oxidative apoptosis associated with p53 and NF-κB pathways.

## 1. Introduction

Alzheimer’s disease is a progressive, neurodegenerative disorder characterized by two important hallmarks: neurotic plaques and neurofibrillary tangles (NFT) [1] meanwhile accompanied by loss of memory and damage of intricate cognition [2].

Fatal component of plaques amyloid-β peptides exert serious neurotoxicity, which causes oxidative stress and cell apoptosis leading to serious consequence [3,4]. Especially the fragment 25-35 of Aβ performs better in regards to solubility and efficiency [5,6] compared to Aβ_1-40_ and Aβ_1-42_, which causes the deficit of memory, neuronal cell apoptosis, synaptic and mitochondrial dysfunction and neuratrophy owing to its special structure [7,8,9,10,11].

Oxidative stress as an early manifestation caught sight of Alzheimer’s disease (AD) has been depicted in passed patients [12]. It causes perturbation between the oxidation and reduction system and decline of mitochondrial membrane potential (MMP), which leads to pro-apoptotic mediators release to initiate cell apoptosis [13]. In mitochondrial apoptosis there is an important balance between B-cell lymphoma-2 (bcl-2) and Bax proteins [14,15,16]. Once this balance is damaged, the caspases activator Cytochrome C will be activated to release into cytosol resulting in caspase 3 activating [17,18]. The nuclear transcription factor p53 [19] and nuclear factor κB (NF-κB), a transcription factor related to inflammation, which exists mostly in the form of heterodimer RelA (p65)/p50 in cytoplasm and translocates important apoptotic gene expressions in the nucleus [20], mediate these protein expressions further [21,22,23]. In view of the above mentioned mechanisms, prevention or inhibition of oxidative stress may be regarded as a key step in AD therapy [24].

Herbal medicines comprising varied components exhibit many pharmacological activities including anti-oxidant, anti-amyloid, and anti-inflammation and so on [25]. *Leontopodium longifolium*, herbal medicine belonged to Asteraceae, widely distributes in Asia and Europe. Previous study has demonstrated that compound *ent*-kaur-15-en-17-al-18-oic acid (LL-3) extracted from it significantly scavenged free radical nitric oxide (NO) that plays an important role in oxidative stress [26], so LL-3 neuroprotective role of SH-SY5Y cells considered as a classical AD cellular model was studied in the current study [27].

## 2. Results

### 2.1. Effect of LL-3 and Amyloid Beta Peptide 25-35 on Cell Viability

**Figure 1:**
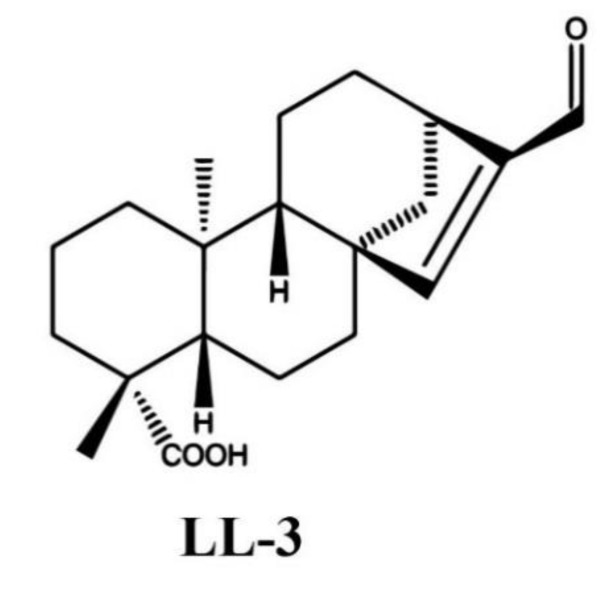
Chemical structure of *ent*-kaur-15-en-17-al-18-oic acid (LL-3).

**Figure 2:**
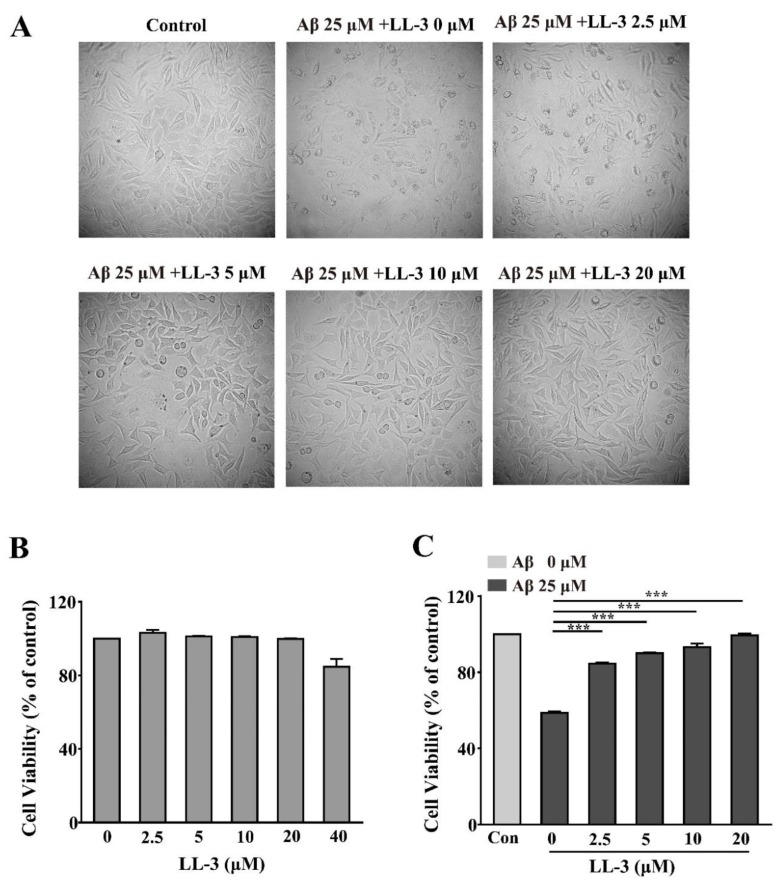
LL-3 protective effect on amyloid beta peptide 25-35 (Aβ_25-35_)-induced neurotoxicity in SH-SY5Y cells. SH-SY5Y cells were treated with different concentrations of LL-3 for 48 h and then cell viability was analyzed according to 3-(4,5-dimethyl-2-thiazolyl)-2,5-diphenyl-2-H-tetrazolium bromide (MTT) assay by measuring the absorbance at 570 nm (**B**). SH-SY5Y cells were treated with different concentrations of LL-3 for 30 min prior to 25 μM Aβ_25-35_ for another 48 h. After this incubation, cell viability was determined using the MTT assay (**C**) and LL-3 co-incubated with Aβ_25-35_ effect on cell viability was viewed under microscope (**A**). All these experiments were detected three times and shown with mean ± SD and *** *p* < 0.001 compared to Aβ_25-35_ alone.

3-(4,5-Dimethyl-2-thiazolyl)-2,5-diphenyl-2-H-tetrazolium bromide (MTT) assay was used to detect LL-3 (chemical structure as shown in Figure 1) [26] cytotoxicity in SH-SY5Y cells. According to Figure 2B, dosage 2.5–20 μM that showed no significant alternation compared to control cells were adopted to detect the LL-3 neuroprotective role in amyloid beta peptide 25-35 (Aβ_25-35_)-induced cytotoxicity in SH-SY5Y cells. As Figure 2C shows, LL-3 reversed the cytotoxic activity caused by Aβ_25-35_. Its neuroprotective role is also viewed under a microscope (Figure 2A). These results suggested that LL-3 may play a part in protecting SH-SY5Y cells from Aβ_25-35_-induced apoptosis in a dose-dependent way.

### 2.2. Effect of LL-3 on Aβ_25-35_-Induced Cellular Reactive Oxygen Species Generation

**Figure 3:**
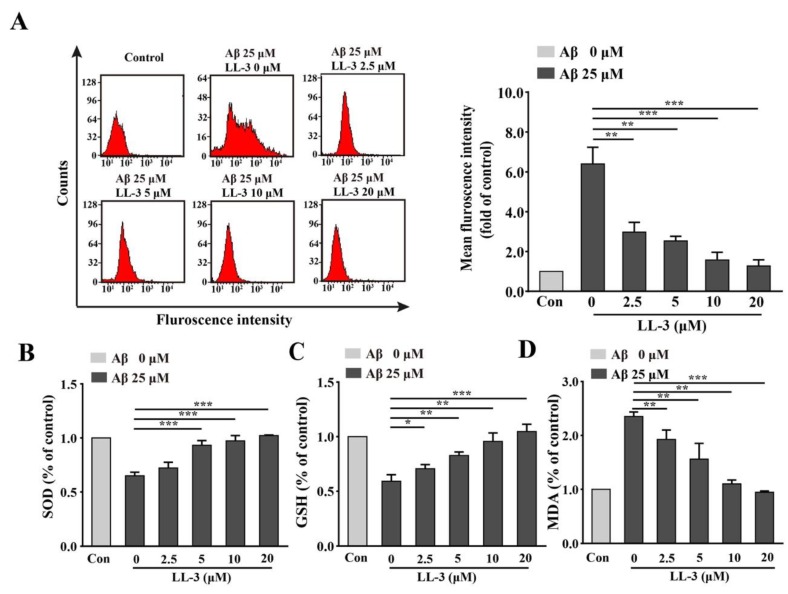
Effects of LL-3 on Aβ_25-35_-induced oxidative stress. SH-SY5Y cells were pretreated with different concentrations of LL-3 for 30 min in six-well plates and co-incubated with 25 μM Aβ_25-35_ for another 24 h. The levels of reactive oxygen species (ROS) (**A**), superoxide dismutase (SOD) (**B**), glutathione (GSH) (**C**), and malondialdehyde (MDA) (**D**) were detected through flow cytometric assay using 2′,7′-dichlorodihydrofluorescein diacetate (DCFH-DA) probe and SOD, GSH, and MDA assays according to manufacturer protocols. All these experiments were detected three times and shown with mean ± SD, * *p* < 0.05, ** *p* < 0.01, *** *p* < 0.001 compared to Aβ_25-35_ alone.

2′,7′-Dichlorodihydrofluorescein diacetate (DCFH-DA) probe that can be hydrolyzed by cellular enzyme to form DCFH. This substance can react with reactive oxygen species (ROS) to produce fluorescent DCF so DCFH-DA probe was used to estimate intracellular ROS production via flow cytometric assay. The assay showed that SH-SY5Y cells treated with Aβ_25-35_ alone caused great elevation of ROS production compared to the control group. LL-3 significantly reduced the ROS level with G-mean at 2.5 μM (0.49), 5 μM (0.44), 10 μM, (0.39), 20 μM (0.31) (Figure 3A), implying that LL-3 may relieve oxidative stress induced by Aβ_25-35_.

### 2.3. LL-3 Decreased Malondialdehyde and Increased Glutathione and Superoxide Dismutase Levels in Aβ_25-35_-Exposed Cells

As important radical cleaners, glutathione (GSH) and superoxide dismutase (SOD) levels were detected in SH-SY5Y cells and the important product of ROS, malondialdehyde (MDA) was also detected in this study to study LL-3 protective role in Aβ_25-35_-induced oxidative stress further. GSH and SOD react with oxyradicals and MDA causes oxidative stress. The results found that the levels of GSH and SOD significantly increased and level of MDA decreased in a dose-independent way through LL-3 co-treatment compared to Aβ_25-35_ alone (Figure 3B–D), suggesting that LL-3 may improve the anti-oxidant system to relive oxidative stress caused by Aβ_25-35_.

### 2.4. LL-3 Protected Aβ_25-35_-Treated Cells MMP from Damage

**Figure 4:**
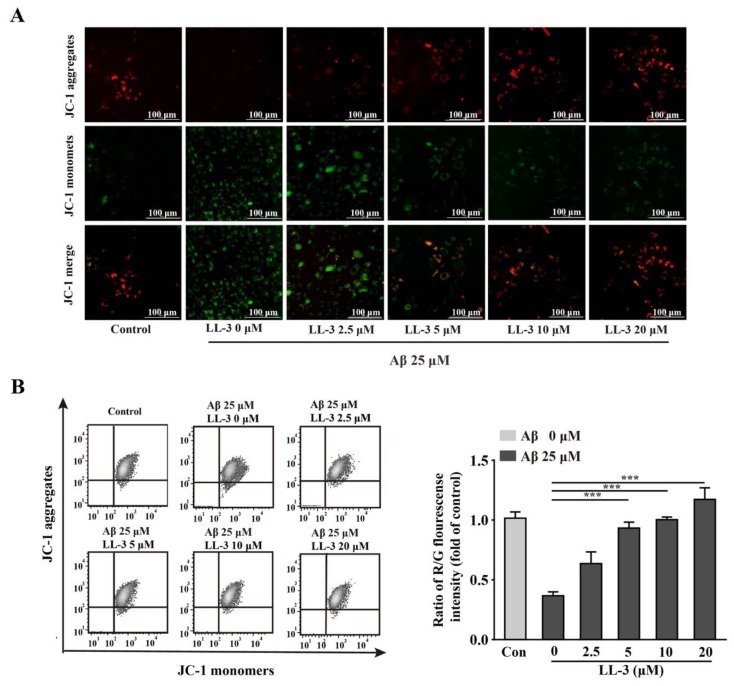
LL-3 recovered mitochondrial membrane potential (MMP) from Aβ_25-35_ damage. MMP, evaluated by JC-1 probe was assessed after LL-3 co-incubation with Aβ_25-35_ for 24 h. LL-3 was pretreated before Aβ_25-35_ for 30 min. (**A**) The MMP alteration was assessed by Cytation 5 Imaging Reader using JC-1 staining. (**B**) The ratio of R/G fluorescence intensity was detected using flow cytometric assay. All experiments were detected three times and shown with mean ± SD and *** *p* <0.001 compared to Aβ_25-35_ alone.

Dysfunction of MMP caused by oxidative stress is also an early and key evidence in apoptotic cells. A JC-1 probe that has two forms with distinct fluorescence is used to detect cellular MMP. Red fluorescence indicates a high concentration of multimer and green fluorescence indicates the monomer pattern. Under oxidative stress conditions, JC-1 is present in the monomer pattern. Cells with exposure to 25 μM Aβ_25-35_ had shown an obvious reduction of MMP with JC-1 probe presenting green fluorescence, indicating that MMP dysfunction induced by oxidative stress happened. Treated with LL-3 (5, 10, 20 μM) obviously ameliorated this undesirable phenomenon (Figure 4) in a dose-dependent way.

### 2.5. LL-3 Prevented Aβ_25-35_-Induced Apoptosis Adopted Diamidino-2-Phenylindole Staining

**Figure 5:**
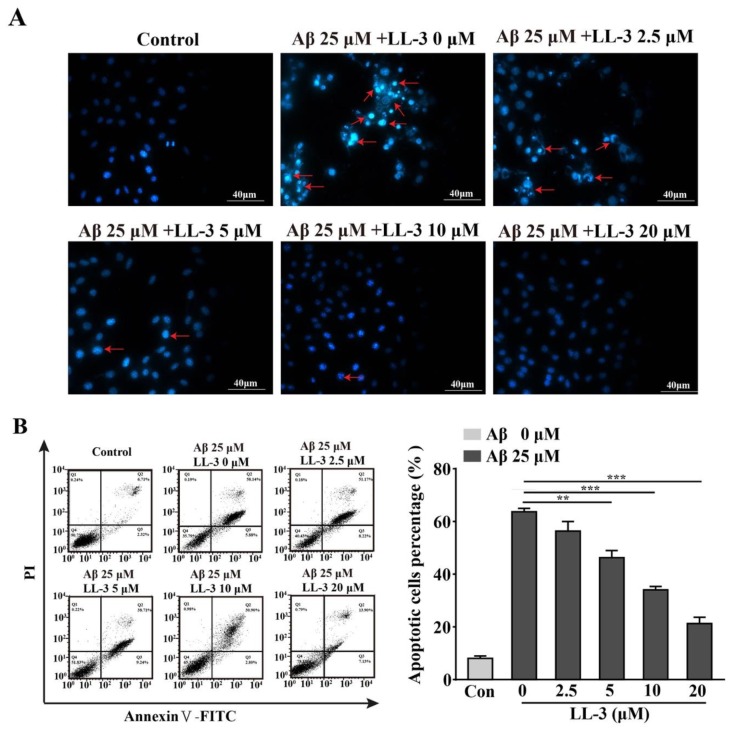
LL-3 inhibited Aβ_25-35_-caused apoptosis. LL-3 was pretreated prior to Aβ_25-35_ for 30 min. After 48 h, cells were detected using diamidino-2-phenylindole (DAPI) staining and viewed under Cytation 5 Imaging Reader. (**A**) Arrows indicated the apoptotic nuclei. (**B**) Quantification of abnormal nuclei after exposure to Aβ_25–35_ in the presence or absence of LL-3. The results are representative of three independent experiments. Data were mean ± SD, ** *p* < 0.01, and *** *p* < 0.001 compared to Aβ_25-35_ alone.

When cells are in mitochondrial apoptotic status, they can form apoptotic bodies including nuclear fragments and organelle which can be stained by diamidino-2-phenylindole (DAPI). In the Aβ_25-35_ treated alone group, massive cells with apoptotic bodies were detected under fluorescent microscope. While exposed to LL-3 (5, 10, 20 μM) this phenomenon can be significantly reversed (Figure 5A).

### 2.6. Effect of LL-3 on Aβ_25-35_-Induced Cellular Apoptosis

Annexin V-fluorexcein isothiocyanate (FITC)/PI staining was adopted to detect cellular apoptosis through flow cytometric assay. FITC detected phosphati-dylserine (PS) moieties flipping outward from inside of cells during early apoptosis exhibiting green fluorescence and PI detected late apoptosis combining with nuclear exhibiting red fluorescence. In the Aβ_25-35_ alone group, quantities of apoptotic cells were largely produced. Exposed to LL-3 reversed this result in a dose-dependent way with apoptotic ratios at 2.5 μM (56.34%), 5 μM (46.27%), 10 μM (34.06%), 20 μM (21.24%) (Figure 5B). All these results suggested that LL-3 may exert a role in protecting cells from apoptosis induced by Aβ_25-35_.

### 2.7. LL-3 Inhibited Aβ_25-35_-Treated Cells from Apoptosis Involved NF-κB and p53 Pathways

**Figure 6:**
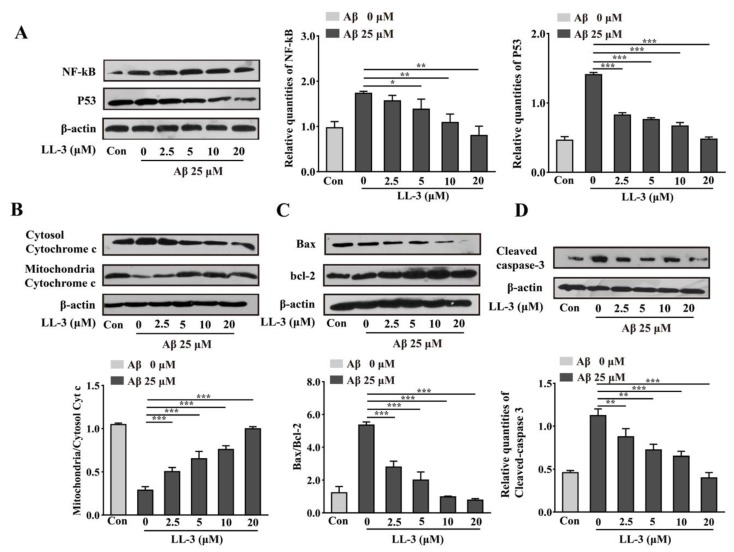
Effect of LL-3 on expressions of NF-κB, p53, bcl-2, Bax, Cytochrome C, and Cleaved-caspase 3 protein through Aβ_25-35_-induced SH-SY5Y cells. Different concentrations of LL-3 were added in cells for 30 min prior to 25 μM Aβ with 48 h. Representative image of western blots of NF-κB, p53 protein (**A**), Cytosol Cytochrome c, Mitochondria Cytochrome c protein (**B**), bcl-2, Bax protein (**C**), Cleaved-caspase 3 protein (**D**). Densitometric analysis of respective changes in levels of values of NF-κB, p53, ratio of Cytosol Cytochrome c/Mitochondria Cytochrome c, ratio of Bcl-2/Bax, and Cleaved-caspase 3. β-actin was used as the internal control. Data were mean ± SD, * *p* < 0.05, ** *p* < 0.01, and *** *p* < 0.001 compared to Aβ_25-35_ alone.

With mitochondrial apoptosis occurring, molecular mechanism was studied using western blot experiment. The Cleaved-caspase 3, bcl-2, Bax, Cytochrome C, p53, NF-κB anti-bodies were applied to detect the compounds molecular mechanism further. Results showed that LL-3 decreased expressions of Bax, Cytochrome C, p53, NF-κB, and cleaved-caspase 3 and increased expression of bcl-2 indicating that compound reversed cellular apoptosis via p53 and NF-κB pathways (Figure 6).

## 3. Discussion

In an important age-related AD symbol, senile plaque which is caused by aggregations of Aβ protein occurs [1]. The accumulations of Aβ cause injury of the brain associated with oxidative stress and cell apoptosis [28]. It is widely accepted that soluble oligomers Aβ triggers the onset of AD mainly through its interaction with brain parenchyma [29,30,31,32]. Aβ_25-35_, short toxic segment corresponding to amino acids 25-35, has full protein encompassing β-sheet [33]. Therefore Aβ_25-35_ has been regarded as vitro model of AD. However, the certain mechanism of AD has not been understood.

Consistent with these reports, a current study found reverse effect of LL-3 on Aβ_25-35_-induced cytotoxicity via MTT assay. According to this, DAPI and Annexin V-FITC/PI methods were used to verify the neuroprotective effect of LL-3 further. Results showed that co-administration of LL-3 can protect cells from apoptosis induced by Aβ_25-35_ in a dose-dependent way.

In the apoptotic process, the bcl-2 family which is defined as two sub-categories: inhibition of apoptosis proteins including primary bcl-2 and bcl-xl maintaining cell survival and another promotion of apoptosis protein represented by Bax, Bid inducing perturbation of mitochondria and cell apoptosis, plays an important role [34]. Activation of Bax and inhibition of bcl-2 induce Cytochrome C that commits cell apoptosis by activating caspase related proteins release. Caspase 3 considered as phenotype of apoptosis which results in maturation of procaspase-3, cleavage of caspase-3 substrates plays an vital role in neuronal apoptosis [35]. Nuclear transcription factor p53 which is also a tumor suppressor may mediate apoptosis in neurons, which can be induced by oxidative stress, DNA damage hypoglycemia, viral infections, and so on. NF-κB a transcription factor can be activated by Aβ deposition to translocate important apoptotic gene expressions in nucleus. Both p53 and NF-κB exert vital roles in mediating these protein expressions. According to this, western blot assay was used to detect apoptotic molecular mechanism further. Aβ_25-35_ exposure leaded to increase expressions of p53, NF-κB, Bax, Cytochrome C, and Cleaved-caspase 3 and decrease expression of bcl-2 but co-administration with LL-3 reversed this phenomenon in a dose-dependent way.

Dysfunction of MMP that causes apoptotic activator Cytochrome C release plays a vital role in increased apoptosis. In agreement with this, JC-1 probe with flow cytometry was used to assess its role. Th Aβ_25-35_ exposed cells showed significantly depletion of MMP while co-administration of LL-3 reversed this grievous phenomenon with elevating MMP in a dose-independent way.

Several studies have affirmed that *Leontopodium longifolium* has anti-inflammation and cough abilities [36]. Persuasive evidences have substantially reported pivotal function of Aβ induced the generation of free radicals which expedite oxidative stress that contributes a lot to process of AD [37,38]. Oxidative stress triggers the onset of mitochondrial apoptosis [39] and indicates a result wherein ROS [40]. ROS, the core resulting in both oxidative stress and decline of MMP also contributes a lot to AD [41]. The superfluous ROS disrupts cellular detoxification through decreasing GSH and SOD levels and augmenting MDA level [41]. Results found that co-administration of LL-3 maintained detoxification system balance through reducing ROS and MDA levels and increasing SOD and GSH levels.

In conclusion, LL-3 inhibited cells from oxidative apoptosis via p53 and NF-κB pathways by maintaining oxidative stress balance, MMP, and inhibiting cell death without obvious cytotoxicity. These findings indicated that LL-3 may become a potential agent for AD therapies.

## 4. Materials and Methods

### 4.1. Preparation of LL-3 Compound

LL-3 (C_20_H_28_O_3_, 316.4345) was produced by our Nature Products Department and spectral data was described previously [26]. LL-3 with the form of purity exceeding 95% colorless crystals was dissolved in the dimethyl sulfoxide (DMSO) as 10 mM, which was diluted in the definite concentration before use. And the control groups were treated with maximum concentration of DMSO alone.

### 4.2. Chemicals

MTT, DAPI, Aβ_25-35_ were purchased from Sigma-Aldrich (St. Louis, MO, USA). DMEM was purchased from Gibco (Gibco, Invitrogen, USA) firm. ROS, SOD, and JC-1 assay kits were purchased from Beyotime Institute of Biotecnology (Shanghai, China). GSH and MDA kits were provided by Nanjing Jiancheng Bioengineering Institute (Nanjing, Jiangsu, China). Annexin V-FITC/PI dyeing kit was from BD Bioscience (San Jose, CA, USA). The anti-bodies of β-actin, p53, NF-κB, bcl-2, Bax, Cleaved-caspase 3, and Cytochrome C were provided by Cell Signaling Technology (CST, Inc, Beverly, MA, USA). The secondary horseradish per-oxidase goat anti-mouse immunglobulin G (IgG) and anti-rabbit IgG were bought from Santa Cruz Biotechnology (Santa Cruz Biotechnology, Inc, Dallas, TX, USA).

### 4.3. Cell Culture

SH-SY5Y (passage 17-30) cells were purchased from the Shanghai Institute Science (SIBS) and were grown in Dulbecco’s modified Eagle medium (DMEM) which contains 10% fetal bovine serum (FBS) (Li Wei Ning, Jinan, China) with 100 units/mL streptomycin and 100 units/mL penicillin at 37 °C in a humidified environment including 5% CO_2_ in cell incubator.

### 4.4. Preparation for Aβ_25-35_ Aggregates

Double distilled water was used to dissolve Aβ_25-35_ with 10 mM concentration that was cultured in cell incubator for seven days to form soluble aggregates. Before use, Aβ_25-35_ aggregates stored at -20°C were diluted to 25 μM.

### 4.5. Determination of Cell Cytotoxicity via MTT Assay

Cells were cultured in 96-well plates at 5 × 10^4^/mL density and treated with different concentrations of LL-3 (2.5–40 or 2.5–20 μM) without or prior to 25 μM Aβ_25-35_ for 30 min. Briefly, after 48 h cells were exposed to 20 μL MTT (5 mg/mL) for 4 h and 150 μL DMSO was used to dissolve crystals. The absorbance was measured with microplate reader (Molecule Device, San Francisco, CA, USA) at 570 nm. The absorbance of control group without LL-3 and Aβ_25-35_ treatments was considered as 100%. All experiments were repeated three times.

### 4.6. ROS Assay

The cultured six-well plates cells were treated with different concentrations of LL-3 (2.5–20 μM) prior to Aβ_25-35_ (25 μM) for 30 min. After 24 h, cells were collected at 1000 rpm for 5 min. Then phosphate buffer saline (PBS) was used to wash cells two or three times. After that cells were loaded with probe at 37 °C for 30 min and mixed it upside down every five minutes. Washed with PBS twice, cells were resuspended with PBS and used flow cytometry (BD Bioscience, San Jose, CA, USA) via FL1-H channel to detect its fluorescence intensity. All experiments were performed three independent times.

### 4.7. Measurement of Intracellular SOD, GSH and MDA Levels

The cells that were cultured in six-well plates were pretreated with different concentrations of LL-3 (2.5–20 μM) for 30 min and then Aβ_25-35_ was added. After 24 h, MDA, GSH, and SOD levels were detected according to manufacturer protocols using microplate reader. All experiments were performed three independent times.

### 4.8. MMP Assay

MMP was measured by flow cytometry using JC-1 probe. Cells were cultured in six-well plates and after indicated treatments, cells were collected at 1000 rpm for 5 min. After this, JC-1 probe was put in cells and co-incubated in 37 °C for 20 min. Then ice-bath JC-1 dyeing buffer washed cells twice. Finally, serum was used to resuspend cells and we detected them via Cytation 5 Imaging Reader (Bio Tek, USA) or flow cytometry through FL2-H and FL1-H channels. Experiments were repeated three times.

### 4.9. Assessment of Morphological Alterations

Six-well plates cells were treated with different concentrations of LL-3 (2.5–20 μM) prior to 25 μM Aβ_25-35_ for 30 min. After 48 h, cells were washed with cold PBS twice and then fixed with ice acetone associated with methanol (1:1 = *v*/*v*) for 5 min. After PBS washed, cells were added in 4 μg/mL DAPI to dye for 10 min in a dark environment. Then Triton-100: PBS (1:1000 = *v*/*v*) was employed to wash cells three times every 5 min. Lastly, anti-fluorescent quenching solution was added in cells. Then apoptotic morphological alteration of cells, which was identified by fragmented or condensed nucleus was detected under Cytation 5 Imaging Reader. Experiments were carried out three times.

### 4.10. Measurement of Cell Apoptosis

Annexin V-FITC/PI dyeing was used to measure cell apoptosis. Cells were cultured in six-well plates. Briefly, post treatment cells as described above were collected at 1000 rpm for 5 min. After washed with PBS, 400 μL binding buffer was added in cells. Then 5 μL Annexin V-FITC was used at 4 °C for 15 min and 10 μL PI was added at 4 °C for another 5 min. Finally, the cells were detected via flow cytometric assay. The proportions about apoptotic cells of total cells were analyzed. Experiments were performed three times.

### 4.11. Western Blot Assay

Cells were cultured in six-well plates. In a word, post treated cells were lysed using RIPA (lysis and extraction buffer) lysate which contains a protease inhibitorphenylmethanesulfonyl fluoride (PMSF) (1 mL RIPA/10 μL PMSF) at 4 °C for 30 min. Then a centrifuge was used to deal with lysate at 12000× *g* for 20 min. Obtained supernatant was used to make protein quantification using BCA kit and western blot assay using 12% SDS-PAGE (polyacrylamide gel electrophoresis) to detect NF-κB, p53, bcl-2, Bax, Cytochrome C, and Cleaved-caspase 3 protein expressions. After being transferred to the NT membrane, apolipoprotein was used to block nonspecific proteins. Following this, corresponding primary antibodies (1:1000) were used to combine with membranous antigens at 4 °C for overnight. Next day, TBST (10 mmol/L Tris-HCL, 150 mmol/L NaCl, and 0.1% Tween-20; pH 7.8) and TBS (150 mmol/L NaCl and 10 mmol/L Tris-HCL; pH 7.8), two wash buffer solution, were used to wash out primary antibodies and secondary horseradish peroxidase goat anti-mouse immunglobulin G (IgG) (1:3000) and anti-rabbit IgG (1:3000) were used to incubate with proteins. Later redundant IgG was washed as described before. Finally, an enhanced chemiluminescence detection system (ECL, Amersham Bioscience) was used to measure target protein expressions. The protein densities were quantified by density software Image J 2.0 (National Institutes of Health, Bethesda, MD, USA). Experiments were performed three times.

### 4.12. Statistical Analysis

GraphPad Prism 7 (Graph-Pad, San Diego, CA, USA) was used to perform statistical analysis and all data are presented as ± SD and * *p* < 0.05, ** *p* < 0.01, or *** *p* < 0.001 are considered as significant difference compared to Aβ_25-35_ alone group using one way ANOVA followed by Tukey’s post hoc test.
